# Expanding the Availability of Onasemnogene Abeparvovec to Older Patients: The Evolving Treatment Landscape for Spinal Muscular Atrophy

**DOI:** 10.3390/pharmaceutics15061764

**Published:** 2023-06-19

**Authors:** Charlotte A. René, Robin J. Parks

**Affiliations:** 1 Regenerative Medicine Program, Ottawa Hospital Research Institute, Ottawa, ON K1H 8L6, Canada; chrene@ohri.ca; 2 Department of Biochemistry, Microbiology, and Immunology, University of Ottawa, Ottawa, ON K1H 8M5, Canada; 3 Centre for Neuromuscular Disease, University of Ottawa, Ottawa, ON K1H 8M5, Canada; 4 Department of Medicine, The Ottawa Hospital, Ottawa, ON K1H 8L6, Canada

**Keywords:** gene therapy, onasemnogene abeparvovec, neuromuscular disease, motor neuron disease, spinal muscular atrophy

## Abstract

Spinal muscular atrophy (SMA) is a devastating neuromuscular disorder caused by mutations in the survival of motor neuron 1 (*SMN1*) gene, which leads to a reduced level in the SMN protein within cells. Patients with SMA suffer from a loss of alpha motor neurons in the spinal cord leading to skeletal muscle atrophy in addition to deficits in other tissues and organs. Patients with severe forms of the disease require ventilator assistance and typically succumb to the disease due to respiratory failure. Onasemnogene abeparvovec is an adeno-associated virus (AAV)-based gene therapeutic that has been approved for infants and young children with SMA, and it is delivered through intravenous administration using a dose based on the weight of the patient. While excellent outcomes have been observed in treated patients, the greater viral dose necessary to treat older children and adults raises legitimate safety concerns. Recently, onasemnogene abeparvovec use was investigated in older children through a fixed dose and intrathecal administration, a route that provides a more direct delivery to affected cells in the spinal cord and central nervous system. The promising results observed in the STRONG trial may support approval of onasemnogene abeparvovec for a greater proportion of patients with SMA.

## 1. Introduction

Spinal muscular atrophy (SMA) is a genetic neuromuscular disorder that was initially described at the end of the nineteenth century by Werdnig [1] and by Hoffmann [2]. SMA is characterized by a degeneration of the alpha motor neurons of the spinal cord, which leads to atrophy of skeletal muscle, although primary defects also occur in many other tissues and organs [3,4,5]. SMA is caused by the homozygous mutation or deletion of the survival of motor neuron 1 (*SMN1*) gene [6], resulting in inadequate levels of survival of motor neuron (SMN) protein within cells [7]. Humans have a paralogous gene, termed *SMN2*, that contains a key C-to-T base transition relative to *SMN1* that induces a skipping of exon 7 in the majority of the messenger RNA (mRNA) transcripts that are derived from this gene, leading to the production of only ~10% full-length SMN protein [8,9]. *SMN2* is variably amplified in humans, and more copies of the gene result in the production of a greater amount of the full-length SMN protein within cells [10]. Thus, more copies of *SMN2* commonly leads to less severe disease for a patient with SMA [11].

Fortunately, several treatments have been approved for this devastating disorder, including nusinersen and risdiplam, both of which promote the inclusion of exon 7 in the *SMN2*-derived mRNA, and thus a greater production of the full-length SMN protein [12,13,14]. Arguably, the most promising therapy, onasemnogene abeparvovec, an adeno-associated virus (AAV)-based vector that encodes the *SMN1* gene, has only been approved for use in infants and young children [15,16,17,18]. Onasemnogene abeparvovec is currently administered intravenously (IV) using a dose based on patient weight, and it is presently unavailable to older children with SMA due to safety concerns related to high absolute doses of vector [15,16,17,18,19]. However, a recent trial has explored the use of administering fixed-dose onasemnogene abeparvovec in children who are up to 5 years of age through intrathecal (IT) administration [20]—a more direct route through which to deliver the therapy to the cells of the spinal cord and central nervous system (CNS). In this review, we will provide background and context for this recent trial.

## 2. Onasemnogene Abeparvovec

Onasemnogene abeparvovec, marketed as Zolgensma, is a self-complementary AAV serotype 9 (scAAV9) vector that encodes a functional copy of *SMN1* that is under the control of a hybrid cytomegalovirus (CMV) enhancer-chicken beta-actin promoter (CBA) [21]. The safety and efficacy of onasemnogene abeparvovec has been explored in several clinical trials, as detailed in Table 1.

Initially, the safety and efficacy of a single IV infusion of onasemnogene abeparvovec was examined in the phase 1 START trial (NCT02122952), which involved symptomatic infants <8 months of age with SMA type 1 and who possessed two copies of *SMN2*. A total of 15 infants received either a low (6.7 × 10^13^ viral genomes (vg)/kg; *n* = 3) or a high (1.1 × 10^14^ vg/kg; *n* = 12) dose of IV onasemnogene abeparvovec. All 15 infants were alive without the need for mechanical ventilation at 20 months of age, a dramatic improvement from the 8% survival rate observed in the historical control cohort. Among 11/12 children in the high-dose cohort, an increase in survival was accompanied by improvements in the Children’s Hospital of Philadelphia Infant Test of Neuromuscular Disorders (CHOP-INTEND) scores compared to their baseline. A total of 13 patients from the START trial were enrolled in a long-term follow-up (LTFU) study (NCT03421977) in which all 10 children from the high-dose group were alive without the need for permanent ventilation and maintained previously achieved motor milestones for up to 7.5 years post-treatment, demonstrating the longevity of onasemnogene abeparvovec [22,23]. Without treatment, children with SMA type 1 with two copies of *SMN2* rarely survive past the age of two, typically succumbing to respiratory failure [24,25].

The follow-up phase 3 trials, STR1VE-US (NCT03306277) and STR1VE-EU (NCT03461289), treated children < 6 months of age with SMA type I and up to two copies of *SMN2* with the high dose used in the START trial [21,26,27]. Both of these trials demonstrated that over 90% of infants survived free of the need for permanent ventilation at 14 months compared to 26% in the natural history cohort; furthermore, approximately half achieved independent sitting at 18 months, a milestone that was not achieved in the natural history cohort. Both STR1VE trials showed a very good benefit–risk profile for the IV administration of onasemnogene abeparvovec in symptomatic infants with SMA that were younger than 6 months, thus supporting the case for the drug’s approval [15]. This favourable benefit–risk profile was also demonstrated in the SPR1NT trial (NCT03505099), which treated pre-symptomatic infants less than 6 weeks of age with 2 (*n* = 14) or 3 (*n* = 15) copies of *SMN2* [28,29]. The START, STR1VE, and SPR1NT trials examined the safety of onasemnogene abeparvovec in both symptomatic and pre-symptomatic infants with SMA; however, all the infants who participated in these studies weighed < 8.5 kg. The Global Managed Access Program (GMAP) launched by Novartis in January 2020 provided treatment to all patients with SMA under the age of 24 months and weighing ≤ 21 kg [30]. As of 2022, 5 out of 102 children who were treated through this program were ≥13.5 kg, the heaviest of which was 17 kg (this patient received a dose of 1.87 × 10^15^ total vg). The GMAP data showed that the safety findings of patients that were ≥8.5 kg at the time of infusion were consistent with the previous data of patients who were <8.5 kg.

As of January 2023, IV onasemnogene abeparvovec was approved in 45 countries [31], although the maximum patient age and/or weight varies between countries. In the United States and Japan, the first countries to approve onasemnogene abeparvovec, the drug is approved for use in children with SMA up to two years old, regardless of weight [15,16]. In Canada and Europe, onasemnogene abeparvovec is used for the treatment of pediatric patients with 5q SMA that have bi-allelic mutations in the *SMN1* gene, and who are either clinically diagnosed as SMA type 1, or possess three or fewer copies of the *SMN2* gene [17,18,32]. This regulatory approval includes dosing guidance for babies and young children up to 21 kg. Based on natural history studies, the majority of untreated children 5 years old or younger with SMA type 1 or type 2 would likely be ≤21 kg, and this could include children as old as 8 years of age [33].

To further explore the efficacy and safety of IV onasemnogene abeparvovec in heavier children, the SMART phase 3b trial (NCT04851873) is investigating the use of IV onasemnogene abeparvovec in children with SMA who were <18 years of age and who weighed ≥8.5 kg to ≤21 kg. Although recruitment criteria for this trial permit participants to be up to 17 years of age, it is unlikely that participants over 8 years of age would meet the weight requirement of ≤21 kg at the time of infusion [33].

Following the success of the STR1VE and SPR1NT trials, there was an obvious desire to examine the efficacy of onasemnogene abeparvovec in an older patient population. However, there are inherent challenges in treating older, and thus larger, patients with SMA. To date, the therapeutic dose has been calculated based on weight at 1.1 × 10^14^ vg/kg. Larger patients would require larger absolute doses of vector. A very high dose of vector is required because IV administration is a relatively inefficient method through which to deliver vector to the CNS—only a small portion of the vector actually transits through the blood–brain barrier (BBB) and transduces the target tissues located within the CNS [34,35]. The majority of the IV-administered vector actually accumulates in the liver and peripheral tissues [36]. No gene therapy vector is completely “safe”; thus, administering a greater quantity of vector raises legitimate safety concerns. Use of AAV, such as onasemnogene abeparvovec, can cause serious adverse events, including acute liver failure in large animal models [34] and in human trials [19], as well as thrombotic microangiopathy (TMA) in human trials [37]—the risk of which increases as more viral genomes are administered. Indeed, trends toward greater elevations in liver transaminases have been reported amongst heavier children who were administered onasemnogene abeparvovec [38,39]; however, this was not the case in all studies [30]. Tragically, two children have died of acute liver failure within 5 to 6 weeks following IV administration of onasemnogene abeparvovec [40,41]. Novartis released a statement following the patient fatalities, highlighting that acute liver failure is a known side effect of onasemnogene abeparvovec; however, the benefit-risk profile is still favourable [41]. Similarly, four children in a clinical trial for the genetic neuromuscular disorder X-linked myotubular myopathy (XLMTM) died following the IV administration of AAV8-MTM1, three of which received a dose of 3.5 × 10^14^ vg/kg and were confirmed to have died from complications of liver failure [42,43,44,45]. AAV may not have been the sole cause of liver failure in these children; it is possible that the administration of the vector could have exacerbated pre-existing liver dysfunction, as intrahepatic cholestasis has been found to be a clinically significant feature of XLMTM [46]. Finally, although the majority of delivered AAV genomes remain episomal, AAV can insert in the cell’s genome, which has been associated with hepatocellular carcinoma in humans [47] and mice [48,49,50] (although not in all studies [51,52,53,54]). In short, although AAV-mediated gene therapy can be very effective, it does not come without risk. For SMA, an alternative approach to achieve efficient delivery to the CNS using less vector is greatly desired.

## 3. Intrathecal Administration of Onasemnogene Abeparvovec

To circumvent the need for the extremely high numbers of vg required for IV treatment in older, and thus larger, patients, IT administration of onasemnogene abeparvovec has been explored with the aim of providing a more efficient transduction of the CNS. In mice and nonhuman primates, IT administration of scAAV9-SMN results in widespread transgene expression throughout the spinal cord, and this is achieved using 10-fold less vector compared to IV administration [55].

Initiated in 2017, the phase 1 STRONG trial (NCT03381729) evaluated a single IT fixed-dose administration of onasemnogene abeparvovec in non-ambulatory children with SMA type 2 and who were 6 to <60-months of age with three copies of *SMN2*. Unlike the previous trials that investigated onasemnogene abeparvovec, there was no weight limit in the eligibility criteria for the STRONG trial. This phase I dose escalation study used doses ranging from 6.0 × 10^13^ to 2.4 × 10^14^ total vg, which is significantly less than the highest dose administered to patients in the IV phase 1 START trial (which administered 1.69 × 10^15^ total vg to the largest patient [21]). Unfortunately, a partial clinical hold was placed on the STRONG trial by the FDA in October 2019, citing concern due to dorsal root ganglia (DRG) mononuclear cell inflammation observed in animals treated with IT onasemnogene abeparvovec [56,57]. Following further long-term preclinical safety studies, the partial clinical hold was lifted in August of 2021 [58].

Recently, results of the completed STRONG trial were published [20]. 32 children were divided into two groups based on age at dosing: younger (6 to <24 months) and older (24 to <60 months). Patients in the younger children grouping were enrolled into one of three fixed-dose cohorts: low- (6.0 × 10^13^ total vg, *n* = 3), medium- (1.2 × 10^14^ total vg, *n* = 13), and high-dose (2.4 × 10^14^ total vg, *n* = 4). The older group all received the medium dose (1.2 × 10^14^ total vg, *n* = 12). Amongst the younger patients, 1/3 (33.3%) in the low-dose cohort and 1/13 (7.7%) in the medium-dose cohort (or 12.5% (2/16 treated patients) when combining the two dosing groups) achieved independent standing. Based on natural history studies, 13.7% would have been predicted to achieve this milestone. Again, amongst the younger children cohort, two children that received the low dose gained a total of four motor Bayley-III milestones (crawls, *n* = 2; pulls to stand, *n* = 1; and stands alone, *n =* 1), while six children in the medium-dose cohort gained a total of 15 motor milestones (rolls, *n =* 4; crawls, *n =* 2; pulls to a stand *n =* 2; stands with assistance, *n =* 3; stands alone, *n =* 1; walks with assistance *n =* 2; and walks alone, *n =* 1). Three children in the high-dose cohort achieved 3 motor milestones (rolls, *n =* 1; pulls to a stand, *n =* 1; and stands with assistance *n =* 1). Older children who received the medium dose (1.2 × 10^14^ total vg, *n =* 12) showed a mean change of 6.0 points in their Hammersmith Functional Motor Scale Expanded (HFMSE) scores at month 12 when compared to baseline, which was significantly different from the natural history cohort, who only saw a 0.5-point change. Furthermore, 11/12 (91.7%) of treated children achieved a clinically meaningful (≥3-point) increase in their HFMSE score compared to their initial score at baseline visit, which is far greater than the 13.3% who achieved a similar improvement in the natural history cohort. Three children in this group achieved a total of four Bayley-III motor milestones (rolling, *n =* 1; standing with assistance, *n =* 2; and walking with assistance, *n =* 1). No deaths were reported in this study. Several adverse events of special interest (AESI) were reported, including nine hepatotoxicity events in seven children (21.9%), including one patient in the medium-dose cohort who experienced elevated alanine aminotransferase (ALT) and aspartate aminotransferase (AST)—which was deemed a treatment-related serious adverse event. There were also five thrombocytopenia events observed in five children (15.6%, one of which was deemed probably treatment related), twelve cardiac events in nine children (28.1%, including one instance each of sinus tachycardia and increased creatinine phosphokinase-MB, which were deemed to possibly be treatment related), and one instance of hepatomegaly (deemed probably treatment related). Overall, the investigators concluded that IT onasemnogene abeparvovec is safe and well-tolerated, and the therapy showed efficacy in children aged 2 to 5 years. Unfortunately, the primary efficacy endpoint of independent standing for ≥3 s in children aged 6 to 24 months was not met during the post-dose observation period of this study.

Patients enrolled in the STRONG trial could participate in LT-002 (NCT04042025), a voluntary phase 4, 15-year follow-up safety and efficacy study, which also enrolled patients from the STR1VE and SPR1NT studies. Novartis provided an update on LT-002 at the 2023 Muscular Dystrophy Association Clinical and Scientific Conference. All children who participated in the STRONG trial had maintained or achieved new developmental gains at a mean follow-up length of 3.6 years [23]. In addition, 5/16 (31.3%) had achieved a new motor milestone; 6 of 12 (50%) had a ≥3-point increase in HFMSE score; and 3 of 12 (25%) had a >10-point increase [59]. However, interpretation of the data is somewhat complicated by the fact that some of the patients also received add-on therapy (nusinersen or risdiplam) [23,59]. Of the 18 patients from the STRONG trial who were enrolled in LT-002, 50% were also receiving add-on therapy [59]. This is far greater than the 24.6% of patients who received add-on therapy following treatment with IV onasemnogene abeparvovec in the STR1VE or SPRINT trials. It is unclear why a larger proportion of patients that received the IT treatment also sought add-on therapy compared to the IV-treated group. Perhaps the systemic distribution of onasemnogene abeparvovec to all tissues of the body during IV treatment provides a better outcome than delivering vector only to the CNS. While combining therapies may be in the best interest of the patient, it does complicate interpretation of the data from the clinical trial as any improvement in motor function may be attributed to more than just onasemnogene abeparvovec. As more data become available, it will be interesting to see if there is a correlation between dose, age, and performance in tests of motor function among those who received add-on therapy compared to onasemnogene abeparvovec alone.

As the medium-dose of IT onasemnogene abeparvovec was well-tolerated and demonstrated efficacy, it was selected for use in the ongoing phase 3 STEER trial (NCT05089656), which is exploring the use of IT fixed-dose onasemnogene abeparvovec in patients aged ≥2 to <18 years old. Unlike the SMART trial—in which patients were only eligible if they were ≤21 kg in weight at the time of infusion—all treated patients in the STEER trial will be administered 1.2 × 10^14^ total vg, regardless of weight. Exploring the efficacy of onasemnogene abeparvovec in the oldest population to date through an IT administration of a fixed dose has the potential to satisfy the unmet need for gene therapy for children and adolescent patients with SMA.

Although IT onasemnogene abeparvovec may be a promising therapeutic option for the treatment of SMA in older children, it is important to note that the most dramatic improvement with IV onasemnogene abeparvovec were observed in pre-symptomatic infants treated at less than 6 weeks of age [28,29]. In the SPR1NT trial, infants with three copies of *SMN2* all achieved independent sitting, and most achieved independent walking, within the normal development window, which is typically not seen in patients with SMA [29]. Amongst infants with two copies of *SMN2*, most sat independently for ≥30 s within the normal development window, and all achieved this milestone by 18 months, while none in the natural history cohort achieved this milestone [28]. In the STRONG trial, children in the younger group gained more Bayley-III motor milestones than those in the older group [20]. Treatment is likely most effective when supplied before the irreversible loss of motor neurons has occurred. These data emphasize the urgent need for universal newborn screening to identify at-risk children as early as possible in order to initiate treatment early. Fortunately, newborn screening has been adopted in many countries around the world [60]. The early treatment of individuals at high risk of developing SMA, prior to symptom onset, results in improved outcomes and greater functional independence, leading to improved quality of life.

## 4. Onasemnogene Abeparvovec versus Nusinersen or Risdiplam

The results of the STRONG trial should be compared to similar trials involving nusinersen, marketed as Spinraza, which is currently available for the treatment of SMA in patients of all ages. Nusinersen is an antisense oligonucleotide (ASO) delivered IT that modulates the splicing of *SMN2* pre-mRNA to promote the inclusion of exon 7, thereby increasing the production of the full-length SMN protein [13,61,62]. As opposed to onasemnogene abeparvovec, which is delivered with a one-time infusion, nusinersen is administered IT through a series of four loading doses over the course of the first two months, followed by maintenance doses every four months [15,63]. All patients receive the same dose (12 mg of the ASO in a 5 mL volume), regardless of age, size, or severity of disease. The CHERISH trial (NCT02292537) used nusinersen to treat 84 children with SMA between 2 and 12 years of age and who had between two and four copies of *SMN2,* with 42 children in a sham control group [64]. At 15 months, 57% of children in the treated group had achieved a change in their HFMSE score of ≥3 points compared to 27% in the sham control group [64]. This is far less than the 91.7% of children who were 2 to 5 years of age and were treated with the medium dose of onasemnogene abeparvovec in the STRONG trial, and who achieved the same increase in HMFSE score at month 12 [20]. Overall, the mean increases in HMFSE scores were not as pronounced in the CHERISH trial at 15 months than in the STRONG trial at 12 months [20,64]. Nevertheless, the CHERISH trial concluded that significant improvement in motor function was observed in children with later-onset SMA compared to the sham control group [64]. Complications associated with lumbar puncture were significantly higher in the treated group than in the control group (15% versus 3%, respectively), and these included back pain, headache, and vomiting. Pyrexia and epistaxis were also ≥5 percentage points higher in the treated group relative to the control group (pyrexia: 43% versus 36%; epistaxis: 7% versus 0%, respectively). This study reported no elevations in ALT or AST as adverse events, unlike in some patients who were treated with onasemnogene abeparvovec [19,20,64].

The use of ASOs such as nusinersen comes with some associated risks, such as renal toxicity, thrombocytopenia, and coagulation abnormalities [63]. Recently, delivery of ASOs into the cerebrospinal fluid (CSF) have been linked to neurotoxicity in mice, which is caused by changes in the intercellular levels of calcium [65]. In several studies in mice, ASO delivery to the CNS has led to behavioural abnormalities, such as decreased desire to explore the cage, reduced responsiveness to stimulation, unusual postures, disrupted breathing patterns, and reduced consciousness [65,66,67,68]. However, this phenomenon has not been reported in any human patients to date.

Importantly, up to 50% of patients in some clinical trials have failed to respond to nusinersen for unknown reasons [64]. Regardless of these drawbacks, nusinersen is considered to be reasonably safe, reasonably effective, and is approved for a wider age group than onasemnogene abeparvovec. Indeed, treatment with nusinersen has demonstrated a stabilization of disease progression and even clinically meaningful improvements in the motor function in some adult patients with SMA [69,70].

Risdiplam, marketed as Evrysdi, is a small molecule splicing modifier approved to treat patients with SMA of all ages [71,72]. It also acts on *SMN2* by stabilizing the *SMN2* transcript and promoting the inclusion of exon 7 to increase the cellular levels of full-length SMN protein [14]. The drug is taken once daily in an oral solution and is dosed based on patient age and weight at 0.15 mg–0.25 mg/kg up to 20 kg, and 5 mg is the maximum recommended dose for all patients above 20 kg [73]. In clinical trials, the use of risdiplam improved HMFSE scores, although to a lesser degree than that observed for nusinersen and IT onasemnogene abeparvovec [20,64,74,75]. Patients between 2 to 11 years of age only achieved a mean 1.7-point increase in HMFSE score at month 24 following treatment with risdiplam [74]. Unlike nusinersen, which is designed to interact with only *SMN2* pre-mRNA, risdiplam does not necessarily target only *SMN2*. Indeed, in animal models risdiplam has been associated with alternative splicing of the off-target genes *FOXM1* and *MADD*, which are associated with the cell cycle and apoptosis, respectively [14]. Whether the off-target splicing of *FOXM1*, *MADD*, or other genes has long-term, adverse consequences in treated patients has yet to be determined. The degree of off-target splicing (if any) in patients has not yet been reported [74,75]. The easy administration of risdiplam makes the drug attractive to patients with SMA; however, the clinical benefits do not appear to be as pronounced as onasemnogene abeparvovec or nusinersen.

## 5. Conclusions

For children with SMA between the ages of 2 and 5, the results of the STRONG trial propose treatment with IT onasemnogene abeparvovec as a therapeutic option as it has a favourable benefit-risk profile. Although there have been some adverse events associated with onasemnogene abeparvovec, this treatment appears more effective than nusinersen in older children with SMA (91.7% versus 57% of children attaining a ≥3-point increase in HFMSE score for IT onasemnogene abeparvovec versus nusinersen, respectively) [20,64]. A ≥3-point change in HFMSE score is considered a clinically meaningful change involving two or three skills [76,77]. Attaining these skills may translate into significant improvements in the quality of life for children with SMA. For example, an improvement in the HFMSE test item “one hand to head in sitting task” may mean that a patient could assist in their feeding or do so independently. IV onasemnogene abeparvovec has been proven to be effective for at least 7 years [22,23]; thus, it is possible the older children who were IT-treated may also maintain achieved motor milestones for many years after treatment. All of the approved treatments for SMA are exceedingly expensive, but a single dose of onasemnogene abeparvovec is reported to be more cost effective than the chronic dosing of nusinersen [78], and thus may ultimately save health care costs.

Some patients with SMA who previously were treated with onasemnogene abeparvovec have started an additional add-on therapy (nusinersen or risdiplam) [23,59]. In fact, there are dedicated clinical trials exploring the safety and efficacy of treatment with either nusinersen (NCT04488133) or risdiplam (NCT05861986) following treatment with onasemnogene abeparvovec. In most cases, add-on therapy is initiated due to a perceived or real plateauing of motor function improvement following the initial onasemnogene abeparvovec treatment [79,80,81]. Results from combinatorial therapies have previously been reported, but these are typically of small sample size or have relatively short follow-up duration [79,80,81]. Onasemnogene abeparvovec, nusinersen, and risdiplam all aim to increase SMN protein concentration, but they use different mechanisms of action to do so [12,13,14,15,16,17,18]. Although the use of multiple drugs will obviously increase the already high cost of treatment, a combinatorial approach to treatment may provide an improved outcome compared to monotherapy for some patients with SMA.

In recent years, the prognosis for SMA has been transformed by the availability of life-changing therapeutics. Most notably, the availability of a gene therapy drug restoring a functional copy of *SMN1* through a single intravenous infusion has allowed infants to not only achieve previously unattainable motor milestones, but to also live far beyond the average lifespan of untreated infants. Now, efforts are focusing on expanding the availability of this transformative drug to other age groups. The results of the STRONG clinical trial investigatingIT administration of onasemnogene abeparvovec in older children provides optimism for the field, and positive outcomes from the STEER trial may support broadening the availability of onasemnogene abeparvovec for patients up to the age of 18. Clinically meaningful improvements in motor function from this and other therapies will hopefully improve the quality of life for a wider group of patients with SMA.

## Figures and Tables

**Table 1:** Onasemnogene abeparvovec clinical trials.

Clinical Trial	Patient Age	*SMN2* Copy #	*n*	Administration Method	Dose	Status *
START Phase 1 NCT02122952					Low: 6.7 × 10^13^ vg/kg High: 1.1 × 10^14^ vg/kg	
	<8 months	2	15	Intravenous		Completed
				
STR1VE-US Phase 3 NCT03306277	<6 months	1 or 2	22	Intravenous	1.1 × 10^14^ vg/kg	Completed
STR1VE-EU Phase 3 NCT03461289	<6 months	1 or 2	33	Intravenous	1.1 × 10^14^ vg/kg	Completed
SPR1NT Phase 3 NCT03505099	<6 weeks	1, 2, or 3	30	Intravenous	1.1 × 10^14^ vg/kg	Completed
STRONG Phase 1 NCT03381729	6–60 months	3	32	Intrathecal	Low: 6.0 × 10^13^ vg Medium: 1.2 × 10^14^ vg High: 2.4 × 10^14^ vg	Terminated
SMART Phase 3b NCT04851873	<17 years	N/A	24	Intravenous	N/A (likely 1.1 × 10^14^ vg/kg)	Active
STEER Phase 3 NCT05089656	≥2–<18 years	N/A	125 (estimated)	Intrathecal	1.2 × 10^14^ vg	Recruiting

* as of June 2023.

## Data Availability

Not applicable.

## References

[1] Werdnig G. (1891). Zwei frühinfantile hereditäre Fälle von progressiver Muskelatrophie unter dem Bilde der Dystrophie, aber anf neurotischer Grundlage. Arch. Psychiatr. Nervenkrankh..

[2] Hoffmann J. (1893). Ueber chronische spinale Muskelatrophie im Kindesalter, auf familiärer Basis. Dtsch. Z. Nervenheilkd..

[3] Yeo C.J.J., Darras B.T. (2020). Overturning the Paradigm of Spinal Muscular Atrophy as Just a Motor Neuron Disease. Pediatr. Neurol..

[4] Deguise M.O., Baranello G., Mastella C., Beauvais A., Michaud J., Leone A., De Amicis R., Battezzati A., Dunham C., Selby K. (2019). Abnormal fatty acid metabolism is a core component of spinal muscular atrophy. Ann. Clin. Transl. Neurol..

[5] Nery F.C., Siranosian J.J., Rosales I., Deguise M.O., Sharma A., Muhtaseb A.W., Nwe P., Johnstone A.J., Zhang R., Fatouraei M. (2019). Impaired kidney structure and function in spinal muscular atrophy. Neurol. Genet..

[6] Lefebvre S., Bürglen L., Reboullet S., Clermont O., Burlet P., Viollet L., Benichou B., Cruaud C., Millasseau P., Zeviani M. (1995). Identification and characterization of a spinal muscular atrophy-determining gene. Cell.

[7] Lefebvre S., Burlet P., Liu Q., Bertrandy S., Clermont O., Munnich A., Dreyfuss G., Melki J. (1997). Correlation between severity and SMN protein level in spinal muscular atrophy. Nat. Genet..

[8] Lorson C.L., Hahnen E., Androphy E.J., Wirth B. (1999). A single nucleotide in the SMN gene regulates splicing and is responsible for spinal muscular atrophy. Proc. Natl. Acad. Sci. USA.

[9] Monani U.R., Lorson C.L., Parsons D.W., Prior T.W., Androphy E.J., Burghes A.H., McPherson J.D. (1999). A single nucleotide difference that alters splicing patterns distinguishes the SMA gene SMN1 from the copy gene SMN2. Hum. Mol. Genet..

[10] Wadman R.I., Jansen M.D., Stam M., Wijngaarde C.A., Curial C.A.D., Medic J., Sodaar P., Schouten J., Vijzelaar R., Lemmink H.H. (2020). Intragenic and structural variation in the SMN locus and clinical variability in spinal muscular atrophy. Brain Commun..

[11] Feldkötter M., Schwarzer V., Wirth R., Wienker T.F., Wirth B. (2002). Quantitative analyses of SMN1 and SMN2 based on real-time lightCycler PCR: Fast and highly reliable carrier testing and prediction of severity of spinal muscular atrophy. Am. J. Hum. Genet..

[12] Singh R.N., Singh N.N. (2018). Mechanism of Splicing Regulation of Spinal Muscular Atrophy Genes. Adv. Neurobiol..

[13] Chiriboga C.A., Swoboda K.J., Darras B.T., Iannaccone S.T., Montes J., De Vivo D.C., Norris D.A., Bennett C.F., Bishop K.M. (2016). Results from a phase 1 study of nusinersen (ISIS-SMN(Rx)) in children with spinal muscular atrophy. Neurology.

[14] Ratni H., Ebeling M., Baird J., Bendels S., Bylund J., Chen K.S., Denk N., Feng Z., Green L., Guerard M. (2018). Discovery of Risdiplam, a Selective Survival of Motor Neuron-2 (SMN2) Gene Splicing Modifier for the Treatment of Spinal Muscular Atrophy (SMA). J. Med. Chem..

[15] AveXis (2019). AveXis Receives FDA Approval for Zolgensma®, the First and Only Gene Therapy for Pediatric Patients with Spinal Muscular Atrophy (SMA). https://www.novartis.com/news/media-releases/avexis-receives-fda-approval-zolgensma-first-and-only-gene-therapy-pediatric-patients-spinal-muscular-atrophy-sma.

[16] Novartis (2020). Novartis Receives Approval from Japanese Ministry of Health, Labour and Welfare for Zolgensma® the Only Gene Therapy for Patients with Spinal Muscular Atrophy (SMA). https://www.novartis.com/news/media-releases/novartis-receives-approval-from-japanese-ministry-health-labour-and-welfare-zolgensma-only-gene-therapy-patients-spinal-muscular-atrophy-sma#:~:text=(%E2%80%9CNovartis%20Pharma%E2%80%9D)%20today,are%20pre%2Dsymptomatic%20at%20diagnosis.

[17] Health-Canada (2021). Summary Basis of Decision—Zolgensma—Health Canada. https://hpr-rps.hres.ca/reg-content/summary-basis-decision-detailTwo.php?linkID=SBD00521#AClinBasisTitle.

[18] European-Medicines-Agency (2020). New Gene Therapy to Treat Spinal Muscular Atrophy. https://www.ema.europa.eu/en/news/new-gene-therapy-treat-spinal-muscular-atrophy-corrected.

[19] Chand D., Mohr F., McMillan H., Tukov F.F., Montgomery K., Kleyn A., Sun R., Tauscher-Wisniewski S., Kaufmann P., Kullak-Ublick G. (2021). Hepatotoxicity following administration of onasemnogene abeparvovec (AVXS-101) for the treatment of spinal muscular atrophy. J. Hepatol..

[20] Finkel R.S., Darras B.T., Mendell J.R., Day J.W., Kuntz N.L., Connolly A.M., Zaidman C., Crawford T.O., Butterfield R.J., Shieh P.B. (2023). Intrathecal Onasemnogene Abeparvovec for Sitting, Nonambulatory Patients with Spinal Muscular Atrophy: Phase I Ascending-Dose Study (STRONG). J. Neuromuscul. Dis..

[21] Mendell J.R., Al-Zaidy S., Shell R., Arnold W.D., Rodino-Klapac L.R., Prior T.W., Lowes L., Alfano L., Berry K., Church K. (2017). Single-Dose Gene-Replacement Therapy for Spinal Muscular Atrophy. N. Engl. J. Med..

[22] Mendell J.R., Al-Zaidy S.A., Lehman K.J., McColly M., Lowes L.P., Alfano L.N., Reash N.F., Iammarino M.A., Church K.R., Kleyn A. (2021). Five-Year Extension Results of the Phase 1 START Trial of Onasemnogene Abeparvovec in Spinal Muscular Atrophy. JAMA Neurol..

[23] Novartis (2023). Novartis Shares Zolgensma Long-Term Data Demonstrating Sustained Durability up to 7.5 Years Post-Dosing; 100% Achievement of All Assessed Milestones in Children Treated Prior to SMA Symptom Onset. https://www.novartis.com/news/media-releases/novartis-shares-zolgensma-long-term-data-demonstrating-sustained-durability-75-years-post-dosing-100-achievement-all-assessed-milestones-children-treated-prior-sma-symptom-onset.

[24] Kolb S.J., Coffey C.S., Yankey J.W., Krosschell K., Arnold W.D., Rutkove S.B., Swoboda K.J., Reyna S.P., Sakonju A., Darras B.T. (2017). Natural history of infantile-onset spinal muscular atrophy. Ann. Neurol..

[25] Finkel R.S., McDermott M.P., Kaufmann P., Darras B.T., Chung W.K., Sproule D.M., Kang P.B., Foley A.R., Yang M.L., Martens W.B. (2014). Observational study of spinal muscular atrophy type I and implications for clinical trials. Neurology.

[26] Day J.W., Finkel R.S., Chiriboga C.A., Connolly A.M., Crawford T.O., Darras B.T., Iannaccone S.T., Kuntz N.L., Peña L.D.M., Shieh P.B. (2021). Onasemnogene abeparvovec gene therapy for symptomatic infantile-onset spinal muscular atrophy in patients with two copies of SMN2 (STR1VE): An open-label, single-arm, multicentre, phase 3 trial. Lancet Neurol..

[27] Mercuri E., Muntoni F., Baranello G., Masson R., Boespflug-Tanguy O., Bruno C., Corti S., Daron A., Deconinck N., Servais L. (2021). Onasemnogene abeparvovec gene therapy for symptomatic infantile-onset spinal muscular atrophy type 1 (STR1VE-EU): An open-label, single-arm, multicentre, phase 3 trial. Lancet Neurol..

[28] Strauss K.A., Farrar M.A., Muntoni F., Saito K., Mendell J.R., Servais L., McMillan H.J., Finkel R.S., Swoboda K.J., Kwon J.M. (2022). Onasemnogene abeparvovec for presymptomatic infants with two copies of SMN2 at risk for spinal muscular atrophy type 1: The Phase III SPR1NT trial. Nat. Med..

[29] Strauss K.A., Farrar M.A., Muntoni F., Saito K., Mendell J.R., Servais L., McMillan H.J., Finkel R.S., Swoboda K.J., Kwon J.M. (2022). Onasemnogene abeparvovec for presymptomatic infants with three copies of SMN2 at risk for spinal muscular atrophy: The Phase III SPR1NT trial. Nat. Med..

[30] Chand D.H., Mitchell S., Sun R., LaMarca N., Reyna S.P., Sutter T. (2022). Safety of Onasemnogene Abeparvovec for Patients With Spinal Muscular Atrophy 8.5 kg or Heavier in a Global Managed Access Program. Pediatr. Neurol..

[31] Novartis (2023). Zolgensma Global Managed Access Program in 2023. https://www.novartis.com/news/zolgensma-global-managed-access-program-2023.

[32] European-Medicines-Agency (2023). Summary of the Risk Management Plan for Zolgensma®.(*Onasemnogene abeparvovec*). https://www.ema.europa.eu/en/documents/rmp-summary/zolgensma-epar-risk-management-plan-summary_en.pdf.

[33] De Amicis R., Baranello G., Foppiani A., Leone A., Battezzati A., Bedogni G., Ravella S., Giaquinto E., Mastella C., Agosto C. (2021). Growth patterns in children with spinal muscular atrophy. Orphanet J. Rare Dis..

[34] Hinderer C., Katz N., Buza E.L., Dyer C., Goode T., Bell P., Richman L.K., Wilson J.M. (2018). Severe Toxicity in Nonhuman Primates and Piglets Following High-Dose Intravenous Administration of an Adeno-Associated Virus Vector Expressing Human SMN. Hum. Gene Ther..

[35] René C.A., Parks R.J. (2021). Delivery of Therapeutic Agents to the Central Nervous System and the Promise of Extracellular Vesicles. Pharmaceutics.

[36] Thomsen G., Burghes A.H.M., Hsieh C., Do J., Chu B.T.T., Perry S., Barkho B., Kaufmann P., Sproule D.M., Feltner D.E. (2021). Biodistribution of onasemnogene abeparvovec DNA, mRNA and SMN protein in human tissue. Nat. Med..

[37] Yazaki K., Sakuma S., Hikita N., Fujimaru R., Hamazaki T. (2022). Child Neurology: Pathologically Confirmed Thrombotic Microangiopathy Caused by Onasemnogene Abeparvovec Treatment for SMA. Neurology.

[38] Waldrop M.A., Karingada C., Storey M.A., Powers B., Iammarino M.A., Miller N.F., Alfano L.N., Noritz G., Rossman I., Ginsberg M. (2020). Gene Therapy for Spinal Muscular Atrophy: Safety and Early Outcomes. Pediatrics.

[39] Weiß C., Ziegler A., Becker L.L., Johannsen J., Brennenstuhl H., Schreiber G., Flotats-Bastardas M., Stoltenburg C., Hartmann H., Illsinger S. (2022). Gene replacement therapy with onasemnogene abeparvovec in children with spinal muscular atrophy aged 24 months or younger and bodyweight up to 15 kg: An observational cohort study. Lancet Child Adolesc. Health.

[40] Silverman E. (2022). Novartis Reports Two Children Died from Acute Liver Failure after Treatment with Zolgensma Gene Therapy. https://www.statnews.com/pharmalot/2022/08/11/novartis-zolgensma-liver-failure-gene-therapy-death/.

[41] Novartis (2022). Zolgensma Acute Liver Failure Update. https://www.novartis.com/news/zolgensma-acute-liver-failure-update.

[42] Astellas (2021). Astellas Reports Update to September 1 Announcement on the ASPIRO Clinical Trial of AT132 in Patients with X-Linked Myotubular Myopathy. https://www.astellas.com/en/news/17161.

[43] Astellas (2020). Audentes Therapeutics Provides Update on the ASPIRO Clinical Trial Evaluating AT132 in Patients with X-linked Myotubular Myopathy. https://www.astellas.com/en/news/22726.

[44] Philippidis A. (2021). Fourth Boy Dies in Trial of Astellas Gene Therapy Candidate. https://www.genengnews.com/news/fourth-boy-dies-in-trial-of-astellas-gene-therapy-candidate/.

[45] Morales L., Gambhir Y., Bennett J., Stedman H.H. (2020). Broader Implications of Progressive Liver Dysfunction and Lethal Sepsis in Two Boys following Systemic High-Dose AAV. Mol. Ther..

[46] Molera C., Sarishvili T., Nascimento A., Rtskhiladze I., Muñoz Bartolo G., Fernández Cebrián S., Valverde Fernández J., Muñoz Cabello B., Graham R.J., Miller W. (2022). Intrahepatic Cholestasis Is a Clinically Significant Feature Associated with Natural History of X-Linked Myotubular Myopathy (XLMTM): A Case Series and Biopsy Report. J. Neuromuscul. Dis..

[47] Nault J.C., Datta S., Imbeaud S., Franconi A., Mallet M., Couchy G., Letouzé E., Pilati C., Verret B., Blanc J.F. (2015). Recurrent AAV2-related insertional mutagenesis in human hepatocellular carcinomas. Nat. Genet..

[48] Zhong L., Malani N., Li M., Brady T., Xie J., Bell P., Li S., Jones H., Wilson J.M., Flotte T.R. (2013). Recombinant adeno-associated virus integration sites in murine liver after ornithine transcarbamylase gene correction. Hum. Gene Ther..

[49] Donsante A., Vogler C., Muzyczka N., Crawford J.M., Barker J., Flotte T., Campbell-Thompson M., Daly T., Sands M.S. (2001). Observed incidence of tumorigenesis in long-term rodent studies of rAAV vectors. Gene. Ther..

[50] Dalwadi D.A., Torrens L., Abril-Fornaguera J., Pinyol R., Willoughby C., Posey J., Llovet J.M., Lanciault C., Russell D.W., Grompe M. (2021). Liver Injury Increases the Incidence of HCC following AAV Gene Therapy in Mice. Mol. Ther..

[51] Bell P., Moscioni A.D., McCarter R.J., Wu D., Gao G., Hoang A., Sanmiguel J.C., Sun X., Wivel N.A., Raper S.E. (2006). Analysis of tumors arising in male B6C3F1 mice with and without AAV vector delivery to liver. Mol. Ther..

[52] Bell P., Wang L., Lebherz C., Flieder D.B., Bove M.S., Wu D., Gao G.P., Wilson J.M., Wivel N.A. (2005). No evidence for tumorigenesis of AAV vectors in a large-scale study in mice. Mol. Ther..

[53] Gil-Farina I., Fronza R., Kaeppel C., Lopez-Franco E., Ferreira V., D’Avola D., Benito A., Prieto J., Petry H., Gonzalez-Aseguinolaza G. (2016). Recombinant AAV Integration Is Not Associated With Hepatic Genotoxicity in Nonhuman Primates and Patients. Mol. Ther..

[54] Sabatino D.E., Bushman F.D., Chandler R.J., Crystal R.G., Davidson B.L., Dolmetsch R., Eggan K.C., Gao G., Gil-Farina I., Kay M.A. (2022). Evaluating the state of the science for adeno-associated virus integration: An integrated perspective. Mol. Ther..

[55] Meyer K., Ferraiuolo L., Schmelzer L., Braun L., McGovern V., Likhite S., Michels O., Govoni A., Fitzgerald J., Morales P. (2015). Improving single injection CSF delivery of AAV9-mediated gene therapy for SMA: A dose-response study in mice and nonhuman primates. Mol. Ther..

[56] Tukov F.F., Mansfield K., Milton M., Meseck E., Penraat K., Chand D., Hartmann A. (2022). Single-Dose Intrathecal Dorsal Root Ganglia Toxicity of Onasemnogene Abeparvovec in Cynomolgus Monkeys. Hum. Gene Ther..

[57] Novartis (2019). Novartis Announces AVXS-101 Intrathecal Study Update. https://www.novartis.com/news/media-releases/novartis-announces-avxs-101-intrathecal-study-update.

[58] Novartis (2021). Novartis Announces Lift of Partial Clinical Trial Hold and Plans to Initiate a New, Pivotal Phase 3 Study of Intrathecal OAV-101 in Older Patients with SMA. https://www.novartis.com/news/media-releases/novartis-announces-lift-partial-clinical-trial-hold-and-plans-initiate-new-pivotal-phase-3-study-intrathecal-oav-101-older-patients-sma.

[59] Connolly A.M., Mercuri E., Strauss K.A., Day J.W., Chien Y.-H., Masson R., Widgerson M., Alecu I., Ballarini N., Mehl L. Intravenous and Intrathecal Onasemnogene Abeparvovec Gene Therapy in Symptomatic and Presymptomatic Spinal Muscular Atrophy: Long-Term Follow-Up Study. Proceedings of the 2023 MDA Clinical & Scientific Conference.

[60] Dangouloff T., Vrščaj E., Servais L., Osredkar D., Group S.N.W.S. (2021). Newborn screening programs for spinal muscular atrophy worldwide: Where we stand and where to go. Neuromuscul. Disord..

[61] Finkel R.S., Mercuri E., Darras B.T., Connolly A.M., Kuntz N.L., Kirschner J., Chiriboga C.A., Saito K., Servais L., Tizzano E. (2017). Nusinersen versus Sham Control in Infantile-Onset Spinal Muscular Atrophy. N. Engl. J. Med..

[62] Passini M.A., Bu J., Richards A.M., Kinnecom C., Sardi S.P., Stanek L.M., Hua Y., Rigo F., Matson J., Hung G. (2011). Antisense oligonucleotides delivered to the mouse CNS ameliorate symptoms of severe spinal muscular atrophy. Sci. Transl. Med..

[63] Biogen (2020). Spinraza Prescribing Information. https://www.spinraza.com/content/dam/commercial/spinraza/caregiver/en_us/pdf/spinraza-prescribing-information.pdf.

[64] Mercuri E., Darras B.T., Chiriboga C.A., Day J.W., Campbell C., Connolly A.M., Iannaccone S.T., Kirschner J., Kuntz N.L., Saito K. (2018). Nusinersen versus Sham Control in Later-Onset Spinal Muscular Atrophy. N. Engl. J. Med..

[65] Jia C., Lei Mon S.S., Yang Y., Katsuyama M., Yoshida-Tanaka K., Nagata T., Yoshioka K., Yokota T. (2023). Change of intracellular calcium level causes acute neurotoxicity by antisense oligonucleotides via CSF route. Mol. Ther. Nucleic Acids.

[66] Southwell A.L., Skotte N.H., Kordasiewicz H.B., Østergaard M.E., Watt A.T., Carroll J.B., Doty C.N., Villanueva E.B., Petoukhov E., Vaid K. (2014). In vivo evaluation of candidate allele-specific mutant huntingtin gene silencing antisense oligonucleotides. Mol. Ther..

[67] Toonen L.J.A., Casaca-Carreira J., Pellisé-Tintoré M., Mei H., Temel Y., Jahanshahi A., van Roon-Mom W.M.C. (2018). Intracerebroventricular Administration of a 2′-O-Methyl Phosphorothioate Antisense Oligonucleotide Results in Activation of the Innate Immune System in Mouse Brain. Nucleic Acid Ther..

[68] Hagedorn P.H., Brown J.M., Easton A., Pierdomenico M., Jones K., Olson R.E., Mercer S.E., Li D., Loy J., Høg A.M. (2022). Acute Neurotoxicity of Antisense Oligonucleotides after Intracerebroventricular Injection into Mouse Brain Can Be Predicted from Sequence Features. Nucleic Acid Ther..

[69] Pechmann A., Behrens M., Dörnbrack K., Tassoni A., Wenzel F., Stein S., Vogt S., Zöller D., Bernert G., Hagenacker T. (2023). Improvements in Walking Distance during Nusinersen Treatment—A Prospective 3-year SMArtCARE Registry Study. J. Neuromuscul. Dis..

[70] Arslan D., Inan B., Kilinc M., Bekircan-Kurt C.E., Erdem-Ozdamar S., Tan E. (2023). Nusinersen for adults with spinal muscular atrophy. Neurol. Sci..

[71] Dhillon S. (2020). Risdiplam: First Approval. Drugs.

[72] Wire B. (2022). FDA Approves Genentech’s Evrysdi (Risdiplam) for Use in Babies under Two Months with Spinal Muscular Atrophy (SMA). https://www.businesswire.com/news/home/20220530005033/en/FDA-Approves-Genentech.

[73] Genentech (2022). Evrysdi Prescribing Information. https://www.gene.com/download/pdf/evrysdi_prescribing.pdf.

[74] Mercuri E., Baranello G., Boespflug-Tanguy O., De Waele L., Goemans N., Kirschner J., Masson R., Mazzone E.S., Pechmann A., Pera M.C. (2022). Risdiplam in types 2 and 3 spinal muscular atrophy: A randomised, placebo-controlled, dose-finding trial followed by 24 months of treatment. Eur. J. Neurol..

[75] Mercuri E., Deconinck N., Mazzone E.S., Nascimento A., Oskoui M., Saito K., Vuillerot C., Baranello G., Boespflug-Tanguy O., Goemans N. (2022). Safety and efficacy of once-daily risdiplam in type 2 and non-ambulant type 3 spinal muscular atrophy (SUNFISH part 2): A phase 3, double-blind, randomised, placebo-controlled trial. Lancet Neurol..

[76] Glanzman A.M., O’Hagen J.M., McDermott M.P., Martens W.B., Flickinger J., Riley S., Quigley J., Montes J., Dunaway S., Deng L. (2011). Validation of the Expanded Hammersmith Functional Motor Scale in spinal muscular atrophy type II and III. J. Child Neurol..

[77] O’Hagen J.M., Glanzman A.M., McDermott M.P., Ryan P.A., Flickinger J., Quigley J., Riley S., Sanborn E., Irvine C., Martens W.B. (2007). An expanded version of the Hammersmith Functional Motor Scale for SMA II and III patients. Neuromuscul. Disord..

[78] Malone D.C., Dean R., Arjunji R., Jensen I., Cyr P., Miller B., Maru B., Sproule D.M., Feltner D.E., Dabbous O. (2019). Cost-effectiveness analysis of using onasemnogene abeparvocec (AVXS-101) in spinal muscular atrophy type 1 patients. J. Mark. Access Health Policy.

[79] Oechsel K.F., Cartwright M.S. (2021). Combination therapy with onasemnogene and risdiplam in spinal muscular atrophy type 1. Muscle Nerve.

[80] Mirea A., Shelby E.S., Axente M., Badina M., Padure L., Leanca M., Dima V., Sporea C. (2021). Combination Therapy with Nusinersen and Onasemnogene Abeparvovec-xioi in Spinal Muscular Atrophy Type I. J. Clin. Med..

[81] Chiriboga C.A., Bruno C., Duong T., Fischer D., Mercuri E., Kirschner J., Kostera-Pruszczyk A., Jaber B., Gorni K., Kletzl H. (2023). Risdiplam in Patients Previously Treated with Other Therapies for Spinal Muscular Atrophy: An Interim Analysis from the JEWELFISH Study. Neurol. Ther..

